# Supplementary material for: Resolving the full spectrum of human genome variation using Linked-Reads
Source: Genome Res. 2019 Apr;29(4):635–45. doi: 10.1101/gr.234443.118 (PMC6442396; doi:10.1101/gr.234443.118)
Supplement: Supplemental Material [file supp_gr.234443.118_Supplemental_longranger-2.2.zip › longranger-2.2.tar.bz2!/lr2.2-public/lib/stan/cmdstan/stan/doc/api/html/vv__vari_8hpp_source.html]

Stan: src/stan/agrad/rev/internal/vv\_vari.hpp Source File


|  |  |
| --- | --- |
|  | Stan  2.5.0  probability, sampling & optimization |


- Main Page
- Related Pages
- Namespaces
- Classes
- Files

- File List
- File Members

All Classes Namespaces Files Functions Variables Typedefs Enumerations Enumerator Friends Macros Pages

- src
- stan
- agrad
- rev
- internal

vv\_vari.hpp

Go to the documentation of this file.

1 #ifndef STAN\_\_AGRAD\_\_REV\_\_INTERNAL\_\_VV\_VARI\_HPP

2 #define STAN\_\_AGRAD\_\_REV\_\_INTERNAL\_\_VV\_VARI\_HPP

3

4 #include <stan/agrad/rev/vari.hpp>

5

6 namespace stan {

7  namespace agrad {

8

9  class op\_vv\_vari : public vari {

10  protected:

11  vari\* avi\_;

12  vari\* bvi\_;

13  public:

14  op\_vv\_vari(double f, vari\* avi, vari\* bvi):

15  vari(f),

16  avi\_(avi),

17  bvi\_(bvi) {

18  }

19  };

20

21  }

22 }

23 #endif

vari.hpp

stan::agrad::op\_vv\_vari::op\_vv\_vari

op\_vv\_vari(double f, vari \*avi, vari \*bvi)

**Definition:** vv\_vari.hpp:14

stan::agrad::vari

The variable implementation base class.

**Definition:** vari.hpp:28

stan::agrad::op\_vv\_vari::avi\_

vari \* avi\_

**Definition:** vv\_vari.hpp:11

stan::agrad::op\_vv\_vari::bvi\_

vari \* bvi\_

**Definition:** vv\_vari.hpp:12

stan::agrad::op\_vv\_vari

**Definition:** vv\_vari.hpp:9


---

[ Stan Home Page ]

*© 2011–2014,
Stan Development Team.*
